# An extensible and unifying approach to retrospective clinical data modeling: the BrainTeaser Ontology

**DOI:** 10.1186/s13326-024-00317-y

**Published:** 2024-08-30

**Authors:** Guglielmo Faggioli, Laura Menotti, Stefano Marchesin, Adriano Chió, Arianna Dagliati, Mamede de Carvalho, Marta Gromicho, Umberto Manera, Eleonora Tavazzi, Giorgio Maria Di Nunzio, Gianmaria Silvello, Nicola Ferro

**Affiliations:** 1 Department of Information Engineering, University of Padova, Padova, Italy; 2 Rita Levi Montalcini Department of Neuroscience, University of Turin, Turin, Italy; 3 Institute of Cognitive Sciences and Technologies, C.N.R, Rome, Italy; 4 Azienda Ospedaliero Universitaria Cittá della Salute e della Scienza, Turin, Italy; 5 Department of Industrial and Information Engineering, University of Pavia, Pavia, Italy; 6 Faculdade de Medicina, Instituto de Medicina Molecular, Universidade de Lisboa, Lisbon, Portugal; 7 IRCCS Foundation C. Mondino in Pavia, Pavia, Italy

**Keywords:** Ontology, Clinical data modeling, Amyotrophic lateral sclerosis, Multiple sclerosis

## Abstract

Automatic disease progression prediction models require large amounts of training data, which are seldom available, especially when it comes to rare diseases. A possible solution is to integrate data from different medical centres. Nevertheless, various centres often follow diverse data collection procedures and assign different semantics to collected data. Ontologies, used as schemas for interoperable knowledge bases, represent a state-of-the-art solution to homologate the semantics and foster data integration from various sources. This work presents the BrainTeaser Ontology (BTO), an ontology that models the clinical data associated with two brain-related rare diseases (ALS and MS) in a comprehensive and modular manner. BTO assists in organizing and standardizing the data collected during patient follow-up. It was created by harmonizing schemas currently used by multiple medical centers into a common ontology, following a bottom-up approach. As a result, BTO effectively addresses the practical data collection needs of various real-world situations and promotes data portability and interoperability. BTO captures various clinical occurrences, such as disease onset, symptoms, diagnostic and therapeutic procedures, and relapses, using an event-based approach. Developed in collaboration with medical partners and domain experts, BTO offers a holistic view of ALS and MS for supporting the representation of retrospective and prospective data. Furthermore, BTO adheres to Open Science and FAIR (Findable, Accessible, Interoperable, and Reusable) principles, making it a reliable framework for developing predictive tools to aid in medical decision-making and patient care. Although BTO is designed for ALS and MS, its modular structure makes it easily extendable to other brain-related diseases, showcasing its potential for broader applicability.

**Database URL** https://zenodo.org/records/7886998.

## Introduction

Automatic Disease Progression Prediction (DPP) is challenging but crucial for better supporting medical practitioners and improving patient quality of life. The training of DPP approaches and automatic decision-making solutions often require large amounts of data, typically unavailable to a single medical centre. The problem is exacerbated for rare diseases, whose rarity and possible short progression make it even more challenging to accrue the amount of data needed to train predictive algorithms [1]. Therefore, in this work, we propose BrainTeaser Ontology (BTO), an ontology explicitly designed to enable uniform data collection and favour data interoperability.

**Challenges.** One of the major challenges when it comes to medical data is data scarcity, especially concerning rare diseases. To mitigate these data interoperability challenges, a viable solution relies on combining retrospective data from multiple centres. Nevertheless, different medical and research centres seldom follow the same data collection procedures. Moreover, the semantics of the retrospective data is rarely the same. To uniform the retrospective data collection procedures and foster the adoption of a common and interoperable semantic framework, the state-of-the-art relies on Ontology-Based Data Access (OBDA) methods [2]. Hence, the main challenges in medical data collection, addressed by BTO are: i) data scarcity, with different research centres having access to only a small number of subjects and data records; ii) data collected and organized in different formats; ii) complex or impossible data interoperability.

In this case, a common ontology is used as an integration layer for the underlying heterogeneous data models and schemas. OBDA methods enable querying, aggregating, and joining large heterogeneous data in a distributed manner using a unique query language. This uniforms the data collection procedures and allows for assigning the same semantics to equivalent information collected by various medical centres. Additionally, as the literature highlights [3], ontologies are a fundamental tool to allow for effective predictive models and automatic decision-making procedures in the medical context. The BRAINTEASER project aims at developing proactive algorithms for the progression of Amyotrophic Lateral Sclerosis (ALS) and Multiple Sclerosis (MS) and, to this end, it needs high-quality data. In the context of the BRAINTEASER project, we developed BTO. The BTO is an ontology tailored to model the anamnestic history and retrospective data of patients affected by two rare brain-related diseases and their clinical progression: MS or ALS. In this sense, BTO provides an open, machine-readable, standardized way to encode clinical information that hospitals and research facilities collect about patients affected by ALS and MS. The two diseases are characterized by a different aetiology: in this sense, they represent the perfect use-case to showcase the flexibility and unifying capabilities of BTO. Our ultimate goal for BTO is to support and guide the data collection and curation procedures, allowing the acquisition of cleaned information that can be seamlessly fed to analysis tools and predictive algorithms.

**Contributions.** BTO allows us to *i)* have a unified model instead of using different and fragmented resources; *ii)* coherently integrate ALS and MS data coming from different medical centers; *iii)* empower data-driven, knowledge-informed Artifical Intelligence (AI) tools for diagnosis and/or progression prediction of ALS and MS diseases. Overall, the BTO can be utilized to offer a unified access point to diverse clinical data concerning ALS and MS diseases. This capability is particularly advantageous in a federated context where multiple medical centers must collaborate and share data modeled according to different schemas and formats. In fact, BTO can serve as a general data model for data integration within Ontology-Based Data Access (OBDA) systems [2]. BTO has the fifth advantage of allowing us to share and re-use the BRAINTEASER Knowledge Base (KB) according to Open Science and Findable, Accessible, Interoperable, Reusable (FAIR) principles. Consequently, BTO allows for improved quality of the medical data available to the community.

As a real-world application, the BTO has been already employed as a common schema to instantiate a KB based on the data provided by multiple data centers within the BRAINTEASER consortium. Moreover, the BTO has also been used to empower various downstream tasks, such as DPP [4, 5], and eXplainable AI (XAI) [6].

BTO was designed following a bottom-up procedure, starting from already available fragmented and heterogeneous retrospective clinical databases from multiple research centers. Hence, the design of BTO is based on the “clinical events” each patient can undergoes, such as onset, diagnosis, visits, clinical tests, treatments, and adverse events. This approach allows for extending the ontology to represent any other clinical event that could be relevant for MS or ALS. Furthermore, the event-based approach enhances ontology re-use as it enables to expand BTO to other rare diseases. In the context of the BRAINTEASER project, the development of BTO plays a fundamental role, as it serves as a unified and comprehensive view of ALS and MS data ensuring they have clear semantics and the desired quality for developing predictive algorithms.

Compared to previous efforts (See “Related work” section), BTO overcomes state-of-the-art by jointly modeling two brain-related diseases and focusing on patients and their clinical history and lifestyle – rather than on biochemical processes associated with brain-related rare diseases as related works do.

The current focus of the BTO is on ALS and MS. Still, the interested practitioner could easily extend the BTO to other rare brain-related diseases by considering the modeling for ALS and MS described in this work as a template.

Finally, we released BTO in Zenodo^1^ in turtle format to promote interoperability, findability, and persistency. Thus, BTO is permanently associated with a Digital Object Identifier (DOI) [7]. BTO has been also uploaded on two repositories for ontologies: BioPortal^2^ and Archivio^3^. In particular, BioPortal automatically linked the classes we defined and annotated in BTO to the corresponding (external) classes of other, authoritative ontologies.

**Outline.** The remainder of this work is organized as follows: “Related work” section reports previous works concerning ontological modeling in the biomedical domain, focusing on neurology. “Methodology” section outlines the methodology and principles followed to design BTO while “The brainteaser ontology” section describes its structure. “Downstream applications” section describes the downstream tasks where BTO has already been used, while “Ontology deployment” section reports some queries that can be run on the KB modeled with BTO. Finally, “Conclusion” section draws some conclusions.

## Related work

Since their conception, ontologies had a crucial role in fostering a common understanding of information structure among people and software agents. Additionally, ontologies allow for computers to access to structured collections of information and rules that can be used to conduct automated reasoning.

A major difference with most of the currently available resources, is the envisioned usage for BTO. The majority of solutions in the ALS and MS domain are thesauri of concepts related to these two diseases, used to annotate unstructured information. BTO serves a different purpose: it is meant as the basis to encode clinical data so that such data can be uniformly accessed in a federated context and interpreted and used in a standardized way.

For instance, a practitioner interested in reproducible and interoperable DPP can use BTO to decide which variables to collect during their clinical study. Then, such variables share the same semantic meaning as those collected by anyone using BTO. Furthermore, it will be possible to access seamlessly the suite of DPP approaches that operate on data represented following BTO either already developed [4, 5], or that will be developed in the future.

The rest of this section provides an overview of previous ontologies and efforts in modelling rare diseases. We also provide a focused analysis on ontologies for modeling ALS and MS. Although some of these efforts share similarities with BTO, their unique features prevent them from being interchangeable with it. For each of such semantic resources, we compare it with BTO in Tables 1, 2 and 3, and 4. More in detail, we report their usage, whether they are available online, if they have been updated recently, their description, and the differences compared to BTO. In terms of usage, we describe if the ontology has been used as a schema for a KB, as a thesaurus (*TH*), or as support for machine learning algorithms (*AI*).

### Ontologies modeling rare or neurological diseases at large

Some efforts have been devoted to modeling multiple neurological diseases at large to obtain a general ontology. Neurological Diseases Ontology (NDO) [8, 9] provides a set of classes to describe neurological diseases, their symptoms, and possible interventions encountered during clinical practice. NDO cannot be used in our specific use case because of multiple reasons. First, aspects related to ALS and MS are addressed in a shallow manner, e.g., there are no classes for questionnaires about the progression of the disease, such as the Revised Amyotrophic Lateral Sclerosis Functional Rating Scale (ALSFRS-R) and Expanded Disability Status Scale (EDSS), nor are available classes to describe specific events such as relapses or pregnancies. Secondly, there are no data properties, making it impossible to encode the pieces of information about patients’ clinical history, which is the objective of BTO. In this sense, as aforementioned, NDO serves as a thesaurus of concepts related to neurological diseases, rather than allowing one to model already available data in a knowledge base. Finally, the NDOWeb Ontology Language (RDF) definition is available, but the URIs of its classes correspond to broken links, thus impairing the ontology usability.

**Table 1:** Ontologies related to BTO that models aspects related to MS or ALS as well as other neurological diseases. Usage *TH* indicates that the ontology is supposed to be treated as thesaurus; *AI* indicates that the ontology is used as additional information for machine learning approaches

[8–10]	Name	Neurological Diseases Ontology (NDO)
	Focus	Neurological Diseases
	Usage	TH
	Online	Partially (Released: 2013 - Last Update: 2014) The ontology is available at https://github.com/addiehl/neurological-disease-ontology/blob/master/src/ontology/ND.owl but the URIs for classes defined within NDO are broken or non-existent.
	Description	Ontology used to describe and annotate medical reports concerning neurologic diseases, including MS and ALS, both in biological terms, but also concerning the course of progression and clinical presentation.
	Differences	Different approach to event modeling. Several relevant aspects of our data, such as pregnancies, onset details and specific questionnaires are missing.
[11]	Name	Holistic Ontology of Rare Diseases (HORD)
	Focus	Rare Diseases
	Usage	TH, AI
	Online	Yes (Released: 2017 - Last update: 2019) https://bioportal.bioontology.org/ontologies/HORD
	Description	Ontology with terminology to annotate tweets about rare diseases, including MS, and enhance machine learning tools used for NLP tasks such as sentiment analysis
	Differences	HORD models only the bio-psico-social state of a person associated with rare diseases. No details about the clinical course of the disease.
[12]	Name	OntoVIP
	Focus	Imaging
	Usage	TH
	Online	Yes (Released: 2013) http://neurolog.i3s.unice.fr/ontoneurolog/v3.0/dolce-particular.owl
	Description	Thesaurus used to annotate medical images
	Differences	Focus only on imaging (included those associated with MS and ALS), no further details on the patient included.

Holistic Ontology of Rare Diseases (HORD) [11] aims at modelling several rare diseases, including MS. In such ontology, the focus is to model information derived from the patients’ social networks regarding their diseases, a specific type of data typically not available to the clinician and therefore not necessary within BTO. Furthermore, HORD does not allow to model treatments, tests, or events the patient undergoes during the progression of the disease.

OntoVIP [12] focuses on modeling and annotating diagnostic and medical images, including those used to diagnose MS or ALS. As for most of the other resources, OntoVIP specializes in a specific type of data, images in this case, and does not model aspects related to the medical history of the patients. Nevertheless, if a clinician needs more fine-grained detail concerning diagnostic imaging, BTO could be easily extended by importing OntoVIP.

### MS ontologies

MS has received far more attention in the ontological research community compared to ALS since it has a more prolonged course and larger prevalence. ﻿Tables 2 and 3 report the main ontologies modeling MS, with details about their usage, availability, and differences from BTO.

**Table 2:** Ontologies related to BTO that model MS aspects. Usage *TH* indicates that the ontology is supposed to be treated as thesaurus, *KB* indicates that the ontology is used as the schema for a knowledge base; *AI* indicates that the ontology is used as additional information for machine learning approaches

[13, 14]	Name	AEDSS Application Ontology
	Usage	AI
	Online	No
	Description	Ontology used to determine automatically the EDSS (AEDSS), using an expert system.
	Differences	Focuses only on EDSS, no other elements of MS modeled.
[15–17]	Name	—
	Usage	AI, KB
	KB Description	240 magnetic resonance images representing white matter lesions, annotated according to the ontology.
	Online	No
	Description	Ontology used to annotate white matter lesions images. Such annotations are further used in a machine-learning algorithm to classify images.
	Differences	Focuses only on white matter lesions and images.
[18]	Name	Multiple Sclerosis Patient Data Ontology (MSPD)
	Usage	KB
	Online	Partially (Released: 2014 – Last Update: 2016). The ontology is available at https://github.com/mark-jensen/mspd/blob/master/mspd_06.owl but the URIs for classes defined within MSPD are broken or non-existent.
	KB Description	Data concerning 10,000 patients and 17,000 follow-up visits from the NYSMSC (not available publicly)
	Description	Extension of NDO [8–10], used to encode self-assessment of the disability perceived by the patients. Such self-assessments are then used to determine the prevalence of different characteristics among the population’s subgroups.
	Differences	MSPD does not consider aspects related to clinical events, besides a set of concepts used to diagnose the MS. BTO, on the other hand, besides diagnostic assays for MS, includes also clinical history details (e.g., previous surgeries, traumas, pregnancies, genetic data).

**Table 3:** Ontologies related to BTO that model MS aspects, part II

[19]	Name	Multiple Sclerosis Ontology (MSO)
	Usage	TH, AI
	Online	Partially (Released: 2014). The ontology is available at https://bioportal.bioontology.org/ontologies/MSO but the MSO for classes defined within MSPD are broken or non-existent.
	Description	Automatically constructed ontology for terms associated with MS to enhance information retrieval models.
	Differences	Thesaurus containing concepts related to the MS and their relation, without focusing on the patient’s clinical history.
[20]	Name	Universal Immune System Simulator (UISS)
	Usage	TH, AI
	Online	No
	Description	Ontology used to describe the specific autoimmune biochemical interactions during MS dynamics to computationally predict its course.
	Differences	UISS focuses mainly on biochemical aspects of the MS and does not consider aspects related to the clinical history of the patients, the most relevant aspect in BTO.
[21]	Name	Symptomatic Treatment of Multiple Sclerosis Ontology (STMSO)
	Usage	TH, KB
	Online	Yes (Released: 2022) https://bioportal.bioontology.org/ontologies/STMSO
	Description	The STMSO is a rich ontology to model the symptomatic treatment of the patients.
	Diffences	STMSO focuses on the symptomatic treatment and is meant to model static data. It does not allow for modelling the clinical history of the patients as a sequence of temporal events (i.e., the medical prescriptions over time). Furthermore, the ontology does not define ranges, domains, constrains, and data types for data and object properties.

Gaspari et al. [13, 14] explored the possibility of computing the *Automatic* EDSS (called AEDSS) employing an underlying ontology. The EDSS is a score describing the disability status of a patient affected by MS and can be computed based on a list of predefined items. Gaspari et al. [14] identified four main ontological classes: the rules used to infer the EDSS scores, the anatomical and functional systems associated with each rule, the questions that allow assessing the degree of impairment, and the overall score. Therefore, the AEDSS Application Ontology is utilized to improve the performance of an expert system in computing EDSS. However, this ontology models only aspects related to the EDSS. Therefore, it is not sufficient to model the entire follow-up and clinical history of a patient affected by MS. Furthermore, it is not currently publicly available.

Esposito and De Pietro [15–17] exploited an ontology to define rules that can be applied to the automatic categorization of images to locate lesions on the brain caused by MS. As for the AEDSS ontology, this ontology considers only a small portion of the data generated during the clinical follow-up of a patient, those related to the imaging. Furthermore, it is not publicly available, impairing its usage.

Jensen et al. [18] developed the Multiple Sclerosis Patient Data Ontology (MSPD) to represent data from patients affected by MS. MSPD is meant to compare the self-assessment made by patients concerning their disability due to MS, with objective criteria and assays done by the clinicians. MSPD focuses exclusively on aspects that strictly concern diagnostic and assays for the MS. BTO, on the other hand, besides these aspects, includes also clinical history details (e.g., previous surgeries, traumas, pregnancies, genetic data).

Malhotra et al. [19] published the Multiple Sclerosis Ontology (MSO), one of the most comprehensive ontologies that model the MS. The Multiple Sclerosis Ontology (MSO) is validated in automatically annotating Electronic Medical Records. MSO does not consider the patient’s clinical history, which is the focus of BTO. In this sense, the MSO is a comprehensive list of terms and concepts related to MS rather than a fully-fledged ontology. Moreover, it does not allow for the description of the procedures, tests, events, and results the patient incurs in.

Pappalardo et al. [20] modeled the UISS, an ontology describing immune system activities. This ontology also includes aspects to simulate underlying MS pathogenesis and its interaction with the host immune system. Similarly to MSO, UISS focuses on the biological mechanisms underlying the MS but does not provide the needed classes and properties to model the patient’s clinical history, which is a requirement of BTO.

More recently, Esfahani et al. [21] defined the STMSO. The ontology is constructed by automatically extracting concepts from a corpus of MS related papers and annotating the concepts reported within being one of the most comprehensive resources concerning MS. There are two main reasons why it cannot be adopted in place of BTO. First, it focuses on the treatment aspect, answering questions such as: “What are the treatments for a given symptom of a person affected by MS”. Secondly, our use case starts from the clinical follow-up of the patients, with temporal occurrences (e.g., visits, relapses, clinical tests) over time. Using STMSO it is impossible to model the sequence of events occurring during the progression of the patient’s disease. Moreover, STMSO’s object and data properties are not fully defined, lacking ranges, domains, constraints, and data types, impairing its adoption in a real-world context.

### ALS ontologies

No specific effort has been devised yet to model the progression of the ALS since, up to now, only the care pathway has been modeled ontologically as shown in Table 4.

**Table 4:** Ontologies related to BTO that model ALS aspects. Usage *TH* indicates that the ontology is supposed to be treated as a thesaurus, *KB* indicates that the ontology is used as a schema for a knowledge base

[22, 23﻿]	Name	OntoPaRON
	Usage	TH, KB
	KB Description	31,260 Annotated ALS “events” (i.e., textual descriptions of occurrences) for 928 patients.
	Online	Partially (Release: 2018 – Last Update: 2020). The ontology is available at https://bioportal.bioontology.org/ontologies/ONTOPARON but the URIs of classes defined within OntoPaRON are broken or non-existent.
	Description	Ontology used to annotate textual data about the care pathway of patients affected by ALS. The objective is a) to study the frequency of specific care actions needed by ALS patients) to determine (frequentistic) relations between different actions.
	Differences	Focuses only on ALS and is mostly French-oriented (terms have their English translation, but French is used for URLs).

Cardoso et al. [22, 23] modeled OntoPaRON, an ontology that focuses on the quality of life and care pathway of ALS patients. Such aspects are not included among BTO’s domain requirements, nor are they typically available among data collected by clinicians that we consider in this work. However, both OntoPaRON and BTO contain a Patient class comprising patient details. Thus, it is possible to extend BTO by linking it to OntoPaRON, if the clinician needs to integrate information about the care pathway.

### Relevant medical thesauri, ontologies, and semantic resources used as a basis for BTO

BTO is based upon several foundation ontologies and semantic resources, such as National Cancer Institute Thesaurus (NCIT), SNOMED-CT, and Unified Medical Language System (UMLS), to ensure homogeneity and compatibility with existing resources. Such relevant semantic resources proved essential to the definition, design, and development of BTO while supplying the entities (re-)used in BTO. Such efforts are reported below.

- National Cancer Institute Thesaurus (NCIT)^4^ [24, 25]: is a public domain thesaurus developed by the National Cancer Institute. Its main objective is to provide clinicians and annotators with codes associated with terminology concepts to annotate documents and ease information retrieval. Its developers, Golbeck et al. [24], state that NCIT is not a full-fledged ontology but is a “nomenclature with ontologic features” as it contains primitive concepts linked with each other.
- SMOMED-CT^5^ [26–28]: Systematised NOmenclature of MEDicine Clinical Terms (SNOMED-CT) is an international clinical reference terminology meant to encode clinical data in a standardized, unambiguous and granular manner.
- ESCO Ontology^6^ [29, 30]: The European Skills, Competences, qualifications and Occupations (ESCO) Ontology encodes ontologically the hierarchy of jobs and occupations identified in the ESCO classification. The multilingual ESCO classification was developed by the European Commission to achieve semantic interoperability throughout Europe.
- ATC Ontology^7^ [31]: The Anatomical Therapeutic Chemical Classification (ATC) Ontology is used to represent ontologically the hierarchy of pharmaceutical substances and their dosage as defined in the ATC Classification.
- OAE^8^ [32]: The Ontology of Adverse Events (OAE) is a community-driven ontology developed to standardize and integrate data relating to Adverse Event occurring subsequently to medical interventions. It is meant to support computer-assisted reasoning. OAE includes 3,000 terms with unique identifiers.
- Pollution Ontology^9^ [33]: Global City Indicator Pollution Ontology developed by the Enterprise Integration Lab, Mechanical & Industrial Engineering, University of Toronto, extends the Foundation Ontology for Global City Indicators to cover Environment Indicators.

Additionally, to standardize BTO, whenever it is possible, components are associated with the corresponding UMLS Concept Unique Identifier (CUI).. Unified Medical Language System (UMLS)^10^ [34] is a repository of biomedical vocabularies developed by the US National Library of Medicine. It has been developed to homogenize names and terms to express the same concept and disambiguate terminologies. UMLS integrates over 2 million names for some 900,000 concepts from more than 60 families of biomedical vocabularies, as well as 12 million relations among these concepts [34]. UMLS CUIs act as a direct gateway to other resources containing equivalent concepts, including SNOMED-CT. BTO adopts NCIT as a reference thesaurus as it shares the similar objective of modeling a specific class of diseases. Nevertheless, we would like to point out that BTO puts in place all the needed measures to ensure that its classes can be mapped to any other standard ontology chosen by the practitioner, allowing them to switch between reference ontologies transparently.

Furthermore, clinicians have developed several standards for defining diseases and related health problems, such as International Classification of Diseases, Version 9 - Clinical Modification (ICD9CM)^11^ [35], International Classification of Diseases, Version 10 (ICD10)^12^ [36], or Medical Dictionary for Regulatory Activities Terminology (MedDRA)^13^ [37]. These are international standards well-known in the medical community. Hence, we can expect physicians to provide annotated data relying on them. Therefore, integrating UMLS concepts in BTO allows for easily mapping this type of information to the thesauri of reference. For instance, one can easily map ICD9CM codes into NCIT Unique Resource Identifiers (URIs) via UMLS concepts.

## Methodology

BTO has been designed exploiting a co-design approach, strictly collaborating with the medical partners and domain experts, to embed their knowledge in BTO and, at the same time, to validate all the design choices. To this end, we operated iteratively, producing several (intermediate) versions of the ontology and discussing them with our domain experts. We exploited the iterative discussion process with the medical partners to ensure that these newly defined concepts correctly described the corresponding real-world concepts and to guarantee the semantic quality of the ontology. BTO models the clinical course and the anamnestic history of patients affected by ALS and MS by exploiting an event-based approach. With “event” we refer to anything that can happen to the patient during their clinical history. For example, at a certain point, the patient will experience an onset: we consider the onset as an event, assign it additional information (e.g., the date, the onset region), and link it to the patient. The subsequent diagnosis, visits, treatments and so on, will be considered events alike. Therefore, each of them will be characterized with a series of additional information and linked to the patient as well. This method provides a unified model instead of using different resources for each disease and it enhances ontology re-use as it is easier to extend BTO to represent other events or other diseases, not needed, or even unknown, at the time of the definition of the ontology.

### Domain requirements

#### Identification of the requirements

To identify the domain requirements and embed in the ontology the experts’ knowledge, we followed a co-design approach. The first phase involved discussing separately with each medical research team from the research centres involved in the BRAINTEASER project. More in detail, the medical research teams are from the hospital of Turin, Italy, and the University of Lisbon, Portugal for ALS and the hospital of Turin, and the IRCCS Foundation Mondino in Pavia, Italy, for MS. In this first phase, we identified the main domain requirements expressed as natural language sentences. The subsequent phase involved aligningÂ the domain requirement of the different research teams by adopting a uniform terminology, identifying common physical-world entities within the natural language descriptions of the domain, and relations between them. The second step involved the usage of actual data provided by the research centres. This allowed us to determine the domain of the various classes, identify shared elements by all research centres, and prepare a first draft version of the BTO. This draft was then validated by the experts in two separate meetings, one specifically focused on ALS and one on MS. Based on the clinician and medical experts’ feedback, we updated the ontology, adding or removing classes when needed. The final step involved the feedback received through the reviews on progressive technical reports – about the development ofÂ the ontology – shared with the various medical teams. Upon reaching a consensus on the domain requirements across all research teams involved in the project, we finalized the definition of the domain requirements, which is reported below.

#### Definition of the domain requirements

As aforementioned, BTO is not designed to encode the semantic knowledge on a specific class of diseases under the form of a thesaurus, but rather it is thought as a means to allow interoperability of the data by encoding it in a KB using an ontology. This allows for different medical and research institutes to collect the data using the same semantics. The core of BTO can be instantiated to encode data from almost any clinical scenario. Nevertheless, it is common for diverse diseases to require different tests, types of interventions, and procedures. To showcase the capabilities of BTO, we instantiate it with the two diseases studied within the BRAINTEASER project, ALS and MS. A practitioner interested in extending BTO to a different disease can adopt an analogous methodology to the one described in the remainder of this manuscript. In a sense, our joint modelling of ALS and MS can be considered as a validation and a showcase of the flexibility and extensibility of BTO.

BTO design is centred on patients and events that can occur during each patient’s clinical history. The patient’s clinical history consists of several events, e.g., occurred traumas, pregnancies, surgical procedures, or treatments. Patient’s clinical course differs among those affected by MS and ALS however, the event-based approach exploited in BTO enables the joint model of the two diseases. Patients’ data requirements are the same for MS and ALS. Therefore, part of BTO is designed to model static variables, e.g., date of birth, biological gender, occupation, and clinical family history. Additionally, several works demonstrate the presence of genetic risk factors for both diseases [38, 39]. Hence, modelling patients’ genomes can enhance the understanding of risk factors for MS and ALS. In addition, pollutant exposure levels, smoking habits, or physical activity can influence the development or progress of both diseases [40, 41].

We provide in the remainder of this subsection an overview of the domain requirements, which revolves around clinical data collection for ALS and MS. A practitioner interested in more specific biochemical details, such as the etiology of the diseases, or biological pathways, can extend BTO, either using a biologic-oriented ontology or with their classes.

##### Multiple sclerosis

MS is an autoimmune disorder mainly affecting young adults characterized by the destruction of myelin in the Central Nervous System (CNS) [42, 43]. Pathologic findings include multiple sharply demarcated areas of demyelination throughout the white matter of the CNS. In terms of clinical manifestations, visual loss, paresthesias, spasticity, loss of sensation, and bladder dysfunction are recurring symptoms [42, 43]. The MS typical pattern consists of recurring attacks, known as relapses, followed by partial recovery. However, acute and chronic progressive forms also occur. More than 2.5 million people currently live with MS worldwide [44]. Given the incidence and impact that ALS and MS have on people’s lives, it is fundamental to devise tools to help clinicians diagnose and treat such diseases.

MS diagnosis is made through a combination of clinical history, neurological examination, and Magnetic Resonance Imagings (MRIs) [45]. In particular, the clinical history of patients affected by MS comprises:

- Cerebrospinal Fluid (CSF) analysis [46];
- The recording of Evoked Potentialss (EPs);
- Clinical Evaluation (e.g., weight and Body Mass Index (BMI) assessments);
- EDSS score [47];
- Hematology Tests.

In addition, MS can manifest itself in different phases, each involving different courses of treatment:

- Clinically Isolated Syndrome (CIS);
- Radiologically Isolated Syndrome (RIS);
- Primary Progressive MS (PP);
- Secondary Progressive MS (SP);
- Relapsing-Remitting MS (RR).

MS is often characterized by a cyclic progression, with periods of worsening of the disease, called relapses and improvements. It is, therefore, of uttermost importance to record symptoms and body areas (sites) involved during relapses. MS relapses are also linked to pregnancies, with a decreased risk of relapses in correspondence with pregnancies, making them an additional important piece of information to be recorded. MS progression is recorded using the EDSS score, which is usually assessed by clinicians during visits. Being able to predict the future EDSS score for each patient can enhance precision medicine. Thus, we record all visits where EDSS is assessed within BTO, to aid the development of predictive models to foresee when the patient will present a worsening condition.

##### Amyotrophic lateral sclerosis

ALS is a heterogeneous neurodegenerative disease associated with motor dysfunction, such as muscle weakness or dysphagia, and cognitive and behavioural changes [48]. ALS affects upper and lower motor neurons in the brain stem and spinal cord [42, 49]. The disease onset usually occurs after age fifty and becomes fatal within three to six years. Clinical manifestations include, among others, progressive weakness, atrophy, hyperreflexia, and the eventual paralysis of respiratory functions. Pathologic features include the replacement of motor neurons with fibrous astrocytes and the atrophy of anterior spinal nerve roots as well as corticospinal [42, 49]. Global estimates indicate that the incidence of ALS ranges between 4.1 and 8.4 per 100,000 persons [50].

The clinical history of patients affected by ALS comprises:

- Anatomical region of the onset (e.g., bulbar or spinal);
- Presence of behavioural or cognitive impairments;
- Pulmonary function tests (e.g., Relative Forced Vital Capacity (FVC) measures);
- Lower vs upper motor neuron predominant phenotype;
- ALSFRS-R rating scale [51];
- Milano-Torino functional staging system (MiToS) functional staging system [52];
- King’s clinical staging method (KINGS) [53].

ALS is characterized by very fast progression requiring a number of medical interventions, with a positive impact on the quality of life of the patients, and prolonging survival, such as the Non-Invasive mechanical Ventilation (NIV) and Percutaneous Endoscopic Gastrostomy (PEG). Being able to predict when a patient will need one of such interventions would allow for preventing medical complications. Thus, we record the occurrence of such events within BTO, to aid the development of predictive models to foresee when the patient will need specific medical interventions.

### Design principles

In the following, we describe how BTO complies with the Open Biological and Biomedical Ontology Foundry (OBO)^14^ and FAIR principles [54]^15^, favoring its adoption in heterogeneous scenarios.

- The ontology is *open* and publicly available. Its definition and description can be found at http://brainteaser.dei.unipd.it/ontology/.
- The ontology schema is defined according to the OWL 1.2 *Common Format*.
- The proposed ontology relies on a unique *URI/Identifier Space* identified by the prefix https://w3id.org/brainteaser/ontology/schema/.
- A description of the *Versioning* procedure, as well as previous versions of BTO, is available as part of the documentation of BTO on the ontology web page.
- The *Scope* of BTO is clearly defined: the ontology is meant to model the anamnestic and clinical history of patients affected by two neurological diseases, ALS and MS.
- Following the OBO principles, we associate *Textual Definitions* to each ontology class, also to favor its re-use in other scenarios.
- Before defining a new relation, *Relations* available on the Relations Ontology (RO) have been considered. None of BTO relations presents the same meaning and could have been replaced with one of the RO – nevertheless, this possibility has always been scrutinized.
- A detailed *Documentation* of the ontology is available on its web page.
- For what concerns *Documented Plurality of Users* and *Commitment To Collaboration*, these aspects are intrinsic in developing and using BTO ontology. Indeed, BTO has been developed in the context of the BRAINTEASER Project, which includes partners from multiple European countries. The co-design approach used to devise BTO defines its *collaborative* nature.
- BTO identifies its *Locus of Authority* into its developers, who are indicated on the web page of the ontology, and in the authors of this paper, that comprises both medical experts in ALS and MS and computer science specialists.
- BTO follows strict *Naming Conventions* described in “Implementation principles” section.
- Finally, the BRAINTEASER consortium is actively working on the *Maintenance* and update of BTO.

#### Validation

BTO has been validated with several online tools to verify its consistency and syntactical validity. The “OOPS! Ontology Pitfall Scanner”^16^ [55] was utilized to confirm the accuracy of this ontology. Furthermore, we validated the ontology using the following tools: the SSN Validation Tool^17^ [56], W3C Resource Description Framework (RDF) Validation Service^18^, and Graphite RDF Triple-Checker^19^. None of the validation tools reported major problems directly linked to BTO. As further evidence of its validity, BTO has been checked from and pushed online on the public repository “Archivio”^20^ [57] where it has been awarded with four stars (the maximum)^21^ for its quality.

### Implementation principles

To provide consistency in BTO some basic principles are adopted when defining classes and properties. These guidelines involve external referencing, annotation properties, and naming conventions.

#### External referencing

Reusing and Referencing external classes is common practice when developing ontologies [58]. Indeed, reusing entities and properties already defined in other resources enforces collaboration and data consistency. External referencing is managed with annotation properties and using the URI of the term in the original thesaurus. Due to its wide adoption and exhaustiveness, our primary choice as the external resource is NCIT [25], but others are also employed when no information is available in NCIT, e.g., Systematised NOmenclature of MEDicine Clinical Terms (SNOMED-CT) or ATC. The choice of NCIT as a main reference resource stems from its widespread adoption [59–62], granting increased interoperability to BTO. If the practitioner is more versed on a different reference resource the mapping between BTO classes and the corresponding classes of other well-known ontologies can be done automatically, as shown for example on the BioPortal page of the ontology^22^. This makes BTO substantially agnostic from the chosen reference ontology.

In particular, external URIs are used when defining named individuals that refer to abstract concepts. On the contrary, when a new class is inserted in BTO, it is defined within the BTO namespace, and connected references are expressed using annotation properties.

#### Namespaces

BTO’s URIs are divided into two namespaces: the schema namespace https://w3id.org/brainteaser/ontology/schema/ and the resource namespace https://w3id.org/brainteaser/ontology/resource/. All URIs corresponding to classes, data properties, and object properties belong to the former namespace, while the latter includes all URIs referring to real-world instances of the entities described in BTO at an ontological level. Notice that, in this sense, the resource namespace is empty until the clinician starts populating it with real-world data. The only instances included in the schema namespace are the named individuals corresponding to Simple Knowledge Organization System (SKOS) concepts (as defined in “Usage of the Simple Knowledge Organization System (SKOS)” section). The choice of including these elements in the schema namespace stems from the fact that akin to relational modelling controlled dictionaries, these entities do not depend on the data underneath but can be seen as a predefined thesaurus of concepts and are a fundamental part of the reality modelled in BTO.

#### Classes definition and annotation properties

All components of BTO have additional information in the form of annotation properties. We defined a list of essential metadata to add when a new class is introduced. Firstly, all classes must have a label denoting the name and a comment, which provides a brief explanation – together with its source (e.g., other thesauri, websites, or textbooks). If the class has an equivalent in NCIT, the name and definition are inherited from the thesaurus. In this case, the class comprises another annotation property called rdfs:isDefinedby expressing the Internationalized Resource Identifier (IRI) corresponding to the NCIT term of reference. Most biomedical vocabularies are mapped in the UMLS^23^ with a unique identifier called CUI [34]. For each class that has a UMLS reference, the annotation property dcterms:conformsTo is instantiated with the URL of the corresponding concept. For the sake of clarity, Table 5 reports all the required annotation properties and their values for the example class bto:Pregnancy.

#### Naming conventions

All components must have a label and a comment. About object properties, BTO uses explanatory labels where the property range is included. In this case, the comment explains the relationship between the two classes involved. Table 6 reports an example of all required information for object properties. Concerning data properties, the label usually includes the name of the domain class so that its meaning is intuitive. A comment with the attribute description and, when available, the definition source are also included. Table 7 reports an example of the required information for data properties, using the property bto:deathCause as an example. Note that, all BTO components can comprise the note annotation property for additional remarks or business logic rules.

### Usage of the Simple Knowledge Organization System (SKOS)

In BTO, external resources have been employed to model the diseases affecting a patient, anatomical sites of traumas, and pharmacological substances. Often we are interested in the abstract concept behind the medical term. When an ontology imports external resources, a modelling pattern is *Classism* [63]. Classism is a design pattern where an external hierarchy is modelled as a hierarchy of ontological classes. In this way, data is stored by instantiating multiple named individuals – all with different URIs – for each class, one for each piece of information of interest. In BTO we avoid classism. Avoiding this approach has two important advantages: *i)* it dramatically reduces the number of required URIs, by not defining multiple named individuals; *ii)* it reduces the complexity of the queries. For instance, if we employ classism to model the anatomical location of patients’ traumas, the query that returns patients who suffered from a head trauma needs to match three triples: one for patients suffering a trauma, one for traumas located in an anatomical location, and one for keeping only anatomical locations of type “Head”. On the other hand, if we avoid classism by defining a unique concept for each anatomical location as a named individual, the above query needs to match only two triples: one for patients suffering a trauma and all traumas located in the head (modelled as the same named individual for all head traumas). Therefore, in BTO, classification schemes that refer to abstract concepts already defined in other semantic resources, are modelled using the SKOS data model^24^. In detail, concepts of hierarchical schemes are modelled as named individuals of type skos:Concept and the relationships among concepts are represented by the object property skos:broaderTransitive. Such property is transitive and asserts that one concept is broader in meaning, i.e. more general, than another. Differently from the rdfs:subClassOf property, skos:broaderTransitive links two named individuals rather than two classes.

**Table 5:** List of required annotation properties. For each class in the Brainteaser Ontology, we define label, comment, isDefinedBy and conformsTo. The table reports the values for the example class “Pregnancy”

Annotation property	Value
rdfs:label	Pregnancy
rdfs:comment	The state or condition of having a developing embryo or fetus in the body (uterus), after union of an ovum and spermatozoon, during the period from conception to birth. [Definition Source: NCI]
rdfs:isDefinedBy	http://purl.obolibrary.org/obo/NCIT_C25742
dcterms:conformsTo	https://uts.nlm.nih.gov/uts/umls/concept/C0032961

**Table 6:** Example of required information for the bto:hasDisease object property. For each object property in BTO, we define a label, a comment describing it, the domain, and the range

Property	Value
rdfs:label	hasDisease
rdfs:comment	It defines the relationship between a person and a disease which he or she suffers from.
Domain	Person
Range	“Disease, Disorder or Finding”

**Table 7:** Example of required information for the bto:deathCause data property. For each data property in BTO, we define the domain,label, and comment the domain, and range

Property	Value
rdfs:label	deathCause
rdfs: comment	The circumstance or condition that results in the death of a living being.
Domain	Patient
Range	rdf:PlainLiteral
dcterms:conformsTo	https://uts.nlm.nih.gov/uts/umls/concept/C0007465

**Fig. 1:**
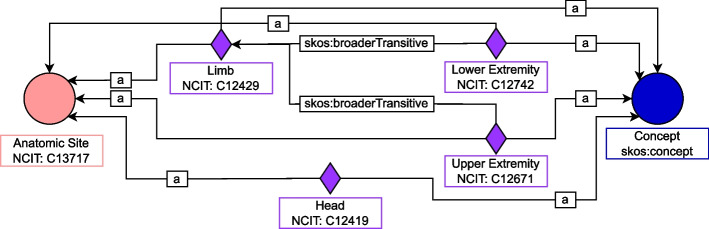
An example of the SKOS data model. Each medical term is modelled as a named individual and the hierarchical scheme is asserted using the object property skos:boraderTransitive. See Table 9 for the legend of the symbols

Figure 1 illustrates this design schema considering the class bto:AnatomicSite as an example. As reported, each body region is modelled as a named individual of type skos:Concept and bto:AnatomicSite, and the terms’ hierarchical structure is asserted using the object property skos:broaderTransitive. For instance, given that limb is a more general concept than upper extremity, the individual representing the abstract concept limb is connected by the above-mentioned property to the one for upper extremity. As a result, the SKOS data model allows for the storage of the location information without instantiating one individual for each patient but by simply referring to the individual already instantiated as a concept. Note that this approach prevents us from describing the peculiarities of the specific entity. However, such a design principle is employed on components that do not have this requirement, i.e. for each class referring to a set of abstract terms without any associated data or object property. Table 8 reports all classes modelled using the SKOS standard and the corresponding semantic resource of reference. NCIT has been employed as the main reference thesaurus whenever it contained the required concepts. We resorted to other well-known resources otherwise. Within BTO namespace, new concepts are defined only if they refer to terms specific to the domain of interest, and the corresponding concept is not available in the considered resources.

**Table 8:** List of classes in the Brainteaser Ontology modeled using the SKOS data model. For each class we specify its name and the reference semantic resource we use to define the concepts

Class	Reference resource
Kinship Type	National Cancer Institute Thesaurus (NCIT)
Occupation	Occupations pillar of the ESCO Classification (ESCO)
Group (social concept)	SNOMED Clinical Terms (SNOMED-CT)
Gene	National Cancer Institute Thesaurus (NCIT)
Disease, Disorder or Findings	National Cancer Institute Thesaurus (NCIT)
Therapeutic Procedure Type	National Cancer Institute Thesaurus (NCIT)
Surgical Procedure Type	SNOMED Clinical Terms (SNOMED-CT)
Anatomic Site	National Cancer Institute Thesaurus (NCIT)
Pharmacologic Substance	Anatomical Therapeutic Chemical (ATC) Classification
Adverse Drug Reaction	Ontology of Adverse Events (OAE)

**Fig. 2:**
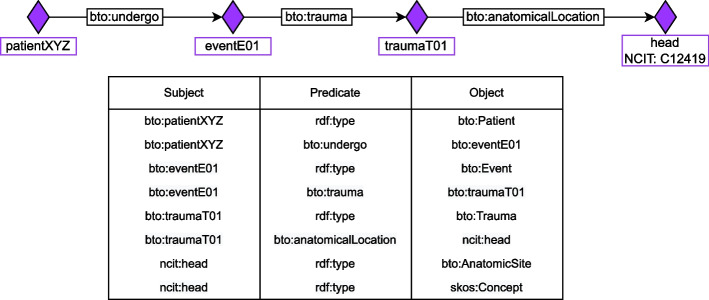
An example of how we model information about a head trauma patient. We show the schema and the individuals involved with a triple table where we report the most important relations. We displayed each triple using the curie notation, particularly, “bto:” stands for elements defined in BTO, “ncit:” refer to the NCI Thesaurus, and “skos:” refers to SKOS namespace. For the sake of readability, we define individual “head” (NCIT:C12419) as ncit:head. See Table 9 for the legend of the symbols

To provide a practical example, assume the clinician needs to model the fact that a patient suffered from head trauma. We do not need to refer to the head of the specific patient – and thus define a URI for it –, but we only need to associate the individual referring to the specific patient’s trauma with a generic individual representing the entity head. Note that we instantiate the specific patient’s trauma and assign a URI to it since we are interested in storing specific information related to each trauma. Indeed, the patient’s trauma has some attributes (such as a date) and might have happened in other places besides the head. Therefore, for all patients affected by head trauma, we create an URI for the specific patient’s trauma, and we link it with the object property bto:anatomicalLocation to the URI of the generic concept of head. The same applies to all head traumas. This example is illustrated in Fig. 2.

## The brainteaser ontology

BTO integrates every aspect that could be useful in understanding the correlation between the disease progression and each patient’s lifestyle or clinical history. These aspects are organized within BTO into eight semantic areas, each denoting a set of ontological classes that refers to a specific aspect of interest in describing the disease progression: “Patient” with section “Environmental Data” (all described in “Patient modeling” section), “Events” (described in “Event modeling” section), “Contingencies” (a more accurate description available in Paragraph “Disease, disorder or finding taxonomy”), and “Intervention or Procedures”, divided into “Surgical Procedures”, “Diagnostic Procedures”, and “Therapeutic Procedures”, respectively detailed in “Intervention modeling” section and Paragraph “Therapeutic procedures modeling”. The ontology description in the remainder of this section focuses on the design choices that we took when developing BTO. Such choices can help a practitioner in adopting or extending the ontology. To avoid encumbering, the complete documentation of BTO, including the technical details, is available at https://brainteaser.dei.unipd.it/ontology/.

**Table 9:**
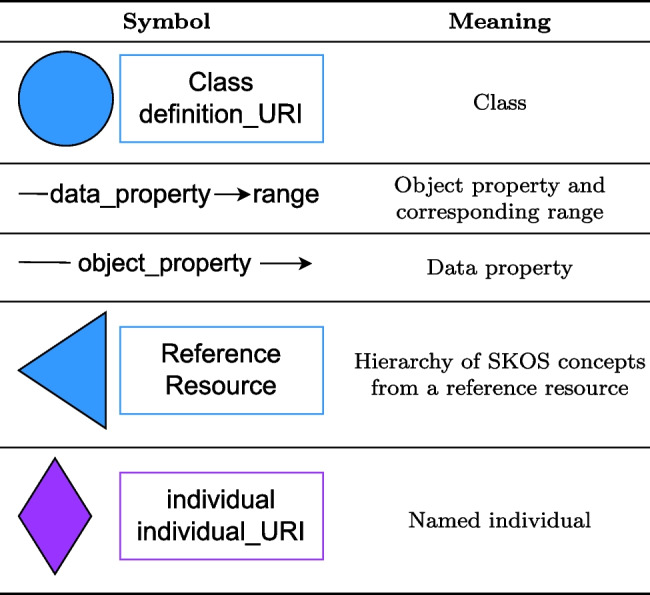
Symbols used in the ontology diagrams

### Patient modeling

**Fig. 3:**
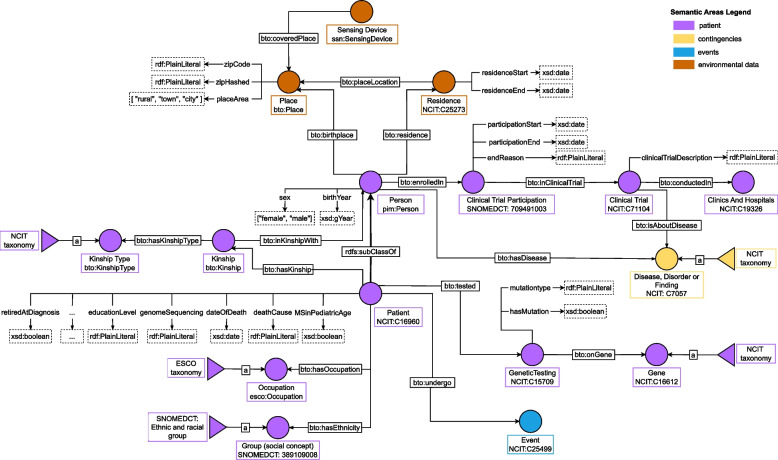
Patient semantic area, identified by the color purple. In particular, yellow specifies the “Contingencies” area’s classes, azure defines events and brown recognizes classes related to environmental information. Patient is a subclass of the class Person from Friend-Of-A-Friend (FOAF ontology. We directly connect to patient data about genetic mutations, occupation, family history, and residence place. The latter is useful to link environmental information to each patient, which is modeled by importing the “Global City Indicator Environment Ontology” [33]. See Table 9 for the legend

Several pieces of information need to be recorded to contextualize the patient’s clinical history better. In this respect, BTO focuses on data about each patient’s lifestyle, clinical events, and family history. Such information is modeled in the “Patient” semantic area, as illustrated in Fig. 3.

Data requirements are similar for patients affected by MS and ALS. In both cases, clinicians need to record personal information, like age and (genotypic) sex, together with clinical family history and possible participation in clinical trials.

bto:Patient is a subclass of bto:Person. This allows us to describe, within the knowledge base, persons who are not patients, such as relatives. In the case of relatives, we want to record that a person, and not a patient, has a certain relationship with the patient and is affected by a given disease. For each patient, information about their relatives is modelled by the class bto:Kinship, which connects patients and relatives with the object properties bto:hasKinship and bto:inKinshipWith, respectively. The property bto:hasKinship links each patient to an instance of the class bto:Kinship, where we can store the degree of relative between a patient and another person. On the other hand, the property bto:inKinshipWith links an instance of the class bto:Kinship to the relative of the considered patient. This modelling choice allows us to specify the different kinship types, e.g. whether we are considering the father or a sister of the patient, which would not be possible if we directly connect each patient to the relative. If we are interested in identifying all patients with a relative affected by ALS, we look for individuals of type bto:Patient connected to nodes of type bto:Kinship. If the relative participating in this relation has object property bto:hasDisease with range ALS (individual NCIT:C34373), then the patient is retrieved. In this case, the individual ALS is a SKOS concept of type bto:DiseaseDisorderOrFinding with IRI from NCIT, according to what we explained in “Usage of the Simple Knowledge Organization System (SKOS)” section. The patients’ disease, i.e. ALS and MS, is modelled using the object property bto:hasDisease which is inherited by the class bto:Person and links each person, or patient, to the disease they suffer from. In this way, we can also store information about each patient’s family history, including relatives’ diseases.

In addition, the clinicians might want to store data about whether a patient’s genome presents specific gene mutations linked to ALS or MS. To encompass this, BTO introduces the “Genetic Data” area. To extract which patients have a gene mutation, one can look for each individual of type bto:Patient that has object property bto:tested and the range node has data property bto:hasMutation set to True. If one also wants to return the gene that presented the mutation, the already extracted individual of type bto:GeneticTesting can be connected with the gene using the object property bto:onGene.

When it comes to describing the patient, BTO shares some aspects with the Common Data Elements (CDEs). The main difference with CDE, is that BTO provides ontological relations between different elements. This has the primary advantage of allowing to adapt and extend BTO with other ontologies if needed. Thanks to the flexibility of the ontologies, including BTO, the interested practitioner can annotate the classes and data properties with the corresponding CDE if needed.

#### Environmental data

**Fig. 4:**
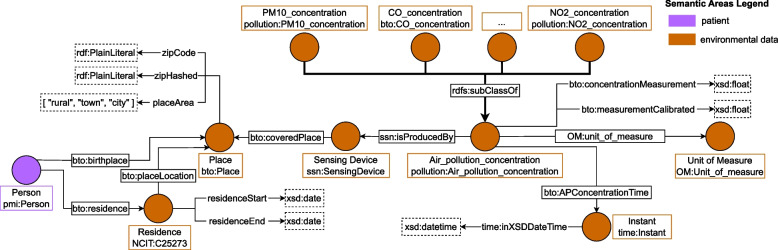
Environmental data integration using the imported “Global City Indicator Environment Ontology” [33]. We linked the class Place with Sensing Device, which produces pollution concentration data. Such information is stored using one of the subclasses of Air Pollution Concentration, representing the specific pollutant. See Table 9 for the legend

Understanding the role of environmental factors can be a great resource for integrating precision medicine into ALS or MS care [64]. Therefore, BTO links environmental data to each patient. These environmental data include pollutant concentrations for a given location recorded by a weather station. To store where the patient lived in BTO, the clinician can record each patient’s birth and residence places using the class bto:Place and the object properties bto:birthplace and bto:residence respectively. Concerning the residence, BTO stores information about the period in which each patient lived in a specific city by means of two data properties namely bto:residenceStart and bto:residenceEnd. To model environmental data, BTO imports *Global City Indicator Environment Ontology*^25^ [33], also known as Pollution ontology, an ontology designed to represent environmental data registered by weather stations. In particular, a weather station, modeled with the class ssn:SensingDevice from the above-mentioned ontology, is connected to a location, modeled with the class bto:Place, with the object property bto:coveredPlace, to model the fact that a station registers environmental data for a given location. Figure 4 reports how air pollutants are integrated into BTO. The concentration of each air pollutant of interest is modelled as a subclass of pollution:Air_pollution_concentration. BTO allows storing information about Particulate Matter <10 $\mu$m (PM10), Particulate Matter <2.5 $\mu$m (PM2.5), Ozone (O3), Nitrogen Dioxide (NO2), Sulphur Dioxide (SO2), and Carbon Monoxide (CO). Air pollution concentrations, i.e. class pollution:Air_pollution_concentration and sensing devices, i.e. class ssn:SensingDevice, are connected with the property ssn:isProducedBy. The concentration value of each air pollutant is stored using two data properties, namely bto:concentrationMeasurement and bto:measurementCalibrated. The former specifies the raw data measured by the sensing device, while the latter defines measured value with seasonal components and noise removed. The date of the measure is referred to with the object property bto:APConcentrationTime that links pollution:Air_pollution_concentration class to class time:Instant. All classes, object properties, and data properties that are not defined within the BTO namespace are imported from the *Global City Indicator Environment Ontology* [33].

### Event modeling

The clinical history of a patient is not directly connected to the bto:Patient class itself, but we assume such data to be registered during a clinical visit where, for instance, the physician fills a form with patient information regarding any relevant clinical event, physical activities, habits, or any clinical condition communicated by the patient to the clinician during the visit. Thus, BTO exploits an event-based design which allows for an easier extension of the ontology to new events of interest or new diseases.

**Fig. 5:**
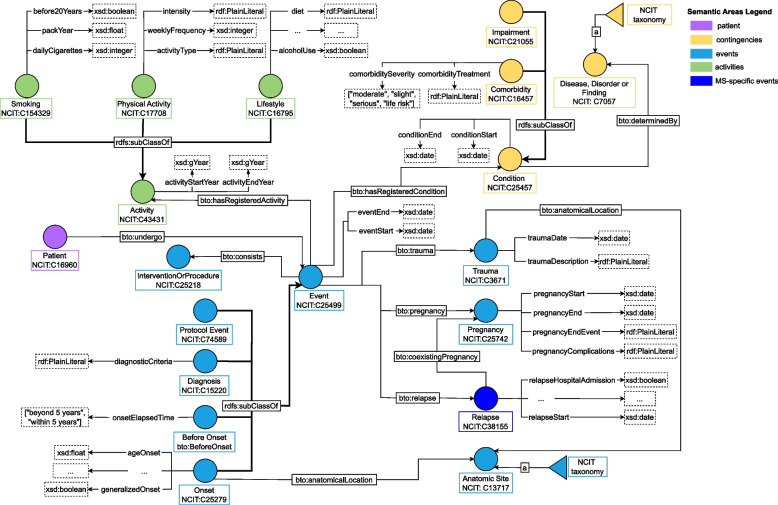
Event semantic area, identified by colour azure. In particular, green specifies the “Activities” semantic area, whereas yellow specifies the “Contingencies” area. We distinguish classes concerning only MS with a darker color, as for the “Relapse” class. The class Event connects each patient to clinical history and lifestyle information. Using such nodes, physicians can store data about habits, past traumas, any clinical event (class “Condition”), coexisting medical conditions (called “comorbidities”), or pregnancies and MS patients’ relapses. See Table 9 for the legend

BTO specifies four subclasses of the class bto:Event, each with its specific data properties: *i)* bto:BeforeOnset, describing events that occurred before the onset of the symptoms and for which there are no specific temporal details; *ii)* bto:Onset, describing the event when a patient experienced the symptoms for the first time; *iii)* bto:Diagnosis event, describing the event and exams carried out when the patient was diagnosed with the disease; *iv)* bto:ProtocolEvent, representing any visit happened after the diagnosis. A visual depiction of the modelling approach is reported in Fig. 5.

In BTO, clinicians can model information about physical activities, smoking habits, and other lifestyle information concerning the patient, by instantiating an individual of type bto:Activity or any of its subclasses: bto:PhysicalActivity, bto:Smoking, and bto:Lifestyle.

For instance, assume the clinician needs to model the fact that, during a check-up, the patient shared information about smoking 10 cigarettes a day. A new individual of type bto:ProtocolEvent is instantiated to record all the details about the check-up. After that, an individual of type bto:Smoking is instantiated and is connected to the event related to the checkup with object property bto:hasRegisteredActivity. Then the frequency information is stored using the data property bto:dailyCigarettes of the smoking node just created. Additional information, such as tests carried out or prescriptions provided during the check-up, will be linked to the bto:ProtocolEvent instance defined above.

Regarding clinical history, during an event, the clinician can register past traumas, coexisting medical conditions, pregnancies, and relapses – in the case of MS patients. These pieces of information constitute the “Contingencies” area, where the class bto:Condition is linked to bto:DiseaseDisorderOrFinding by the object property bto:determinedBy. For instance, if clinicians need to model the fact that a patient had flu–like symptoms, they instantiate an individual of type bto:Condition and add a triple stating that, during the considered event, a condition determined by flu–like symptoms (individual NCIT:C78302) was recorded. Note that the bto:Condition class is used to model each patient’s clinical history, i.e. past traumas, symptoms, or comorbidity, while the disease each patient suffers from is modelled with the object property bto:hasDisease, linking each patient directly to the class bto:DiseaseDisorderOrFinding.

#### Disease, disorder or finding taxonomy

The class bto:DiseaseDisorderOrFinding includes diseases, like carcinoma or chickenpox infection, but also injuries, symptoms, and findings. This allows modeling and storing any sort of clinical event that occurred to a patient, even if it is not directly linked to ALS or MS.. This class is modeled following the SKOS data model illustrated in “Usage of the Simple Knowledge Organization System (SKOS)” section, with NCIT as reference taxonomy and a few additions from the SNOMED-CT thesaurus.

As illustrated in Fig. 5, past traumas are modeled with the class bto:Trauma. However, some injuries have also been made available in the bto:DiseaseDisorderOrFinding taxonomy. As a general rule, we use the taxonomy whenever the trauma is specific (e.g., shoulder dislocation, modeled with individual NCIT:C35020). On the other hand, if the clinician knows only that a patient suffered from generic trauma to the head, an individual of type bto:Trauma is instantiated and connected to its Anatomic Site (e.g., head, modelled with individual NCIT:C12419) with the property bto:anatomicalLocation. Notice that, thanks to SKOS characteristics, specific traumas (e.g., shoulder dislocation) are in relation skos:broaderTransitive with the more general concept injury (NCIT:C3671).

BTO follows a similar line of thought to deal with symptoms. In this case, the taxonomy is used to model cases where the clinician needs to store information about general symptoms (e.g., headache, fever).

A particular group of symptoms that required deeper modelling granularity is the one that constitutes the onset of the disease (either ALS or MS). Given the high relevance that such symptoms have on the course of the disease, they have not been modelled using the symptoms taxonomy but as data properties of the class bto:Onset.

### Intervention modeling

**Fig. 6:**
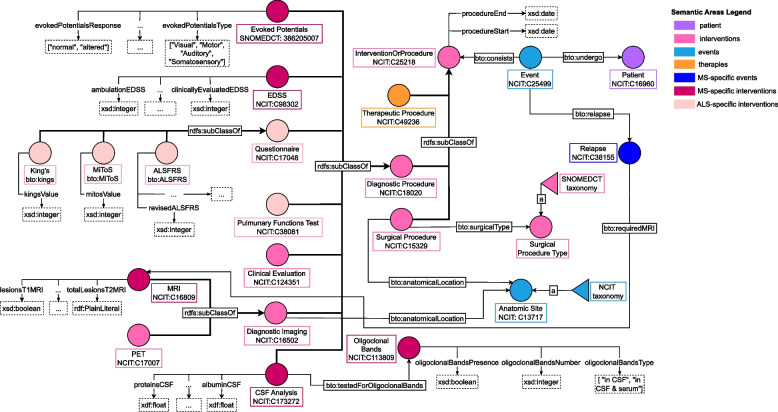
“Intervention or Procedures” area, identified by the color pink. Classes in orange determine the “Therapeutic Procedures” area while we distinguish classes related to a specific disease with different colour tones. In particular, dark pink identifies classes related to MS whereas light pink ALS-specific classes. Class “Event” has 3 subclasses: surgical procedures, diagnostic procedures, and therapeutic procedures. Both surgical and therapeutic interventions are the same for MS and ALS patients while diagnostic procedures can differ based on the disease. See Table 9 for the legend

According to the proposed modelling paradigm, an event might include one or more interventions. For instance, a visit might include multiple exams and multiple prescriptions of therapeutic substances. Therefore the “Intervention or Procedures” semantic area is divided into three subareas: surgical procedures, diagnostic tests, and therapeutic treatments. Figure 6 illustrates the main classes and properties involved in this semantic area. bto:InterventionOrProcedure has 3 subclasses: bto:SurgicalProcedure, bto:DiagnosticProcedure and bto:TherapeuticProcedure.

#### Surgical procedures modeling

The class bto:SurgicalProcedures includes all treatments that involve surgery. Since different surgery procedures might vary widely, BTO defines the class bto:SurgicalType to represent different surgery types. This class is modelled following the SKOS data model and using the “procedure” subhierarchy of SNOMED-CT as the main reference. Following the design pattern used for traumas, if the clinician does not have specific information about the surgery other than its location, it is possible to instantiate the patient’s surgery and link it to an abstract concept representing the anatomical location. For instance, if the clinician wants to record that the patient had a thoracic surgery we instantiate a new node of type bto:SurgicalProcedure with object property bto:anatomicalLocation and range NCIT:C12799 (i.e., thorax). Indeed, the bto:SurgicalProcedure class is linked to bto:AnatomicSite allowing clinicians to store information about which body region was affected by the surgery. As a result, BTO allows the storage of the surgery type or the anatomical location based on the information available or the need of the developed application.

#### Diagnostic procedures modeling

Diagnostic procedures differ based on MS or ALS patients. For instance, MS patients can take an EP test or a CSF analysis and their level of disability can be monitored over time using the EDSS score. On the contrary, ALS patients are tested on their pulmonary function and the disease progression can be assessed using the ALSFRS-R, KINGS, or MiToS scores. Diagnostic intervention information can be a great resource to assess the disease progression of each patient. For example, BTO can return the number of patients having an EDSS score in a specific range, which can be an indicator of impairment severity in patients affected by MS. One peculiarity is that haematology test components are modelled with a class called bto:LaboratoryTestResult where the clinician can specify the component name, the measured level, its unit of measure, and whether the result was normal. BTO also includes a data property called bto:positivyResult to express the presence or absence of specific blood components (e.g., antibodies). This modelling choice allows flexibility, as any blood test result can be included, rather than limiting it to a predefined set of components.

#### Therapeutic procedures modeling

**Fig. 7:**
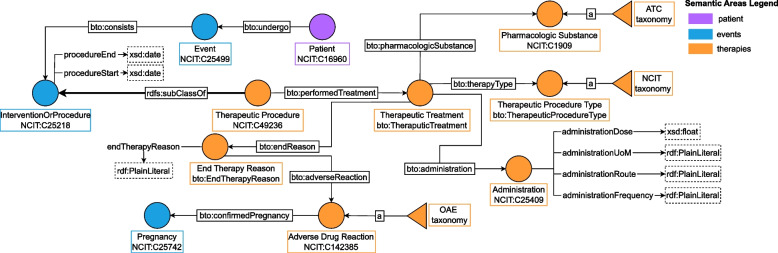
“Therapeutic Procedures” area. The core of the area is the Therapeutic Treatment class, where one can store information about drug administration and posology. In particular, the End Therapy Reason is modelled as a class to link possible causes, such as adverse events and pregnancy. See Table 9 for the legend

BTO identifies a specific semantic area for “Therapeutic Procedures”, which comprises any treatment and the administration of pharmacologic substances. The clinician could administrate multiple pharmacologic substances in a single therapeutic treatment in different time periods. For this reason, BTO introduces the class bto:TherapeuticTreatment, which is the core of the semantic area illustrated in Fig. 7. This class models drug administrations and other therapeutic treatments, e.g. NIV. The class bto:TherapeuticProcedureType is used to specify the pharmacologic substance category or the type of therapy, if not pharmacological. For instance, if clinicians need to record information about a patient requiring NIV one can create a new node of type bto:TherapeuticTreatment with object property bto:therapyType and range NIV (individual NCIT:C171457). With this model, clinicians can store information about the administration specifics like dose and frequency and they can also insert data about the reason for the interruption of treatment. In such case, the class bto:EndTherapyReason can be connected to the class bto:AdverseDrugReaction or bto:Pregnancy. We can also store information about other causes of interruptions by means of the data property bto:endTherapyReason. Concerning therapeutic treatments, adverse drug reactions are modeled as SKOS hierarchies using the OAE Ontology, pharmacological substances using the ATC Ontology, and therapeutic procedure type relying on NCIT.

## Downstream applications

BTO has already been integrated into different downstream applications: Disease Progression Prediction (DPP) and eXplainable AI (XAI). About DPP, the BTO served as the reference ontology for the KB encoding the BRAINTEASER data, from which the datasets used in the intelligent Disease Progression Prediction Challenge at CLEF (iDPP@CLEF) were extracted. iDPP@CLEF challenges provide real patient clinical data on ALS and MS diseases, aiming to foster the development of tools able to support clinicians in all the phases of the patient treatment, suggest personalized therapeutic decisions, and promptly indicate required interventions. Designed together with medical experts from the research centres involved in the BRAINTEASER project, these challenges represent the first step towards the use of BTO in the clinical practice.

In the following, we show how the model provided by BTO encodes essential information for iDPP@CLEF datasets. At the time of writing, iDPP@CLEF has been run three times. In 2022 (first edition), the challenge focused on ALS [4, 5] while in 2023 and 2024 tasks involved both ALS and MS [65–69].

iDPP@CLEF 2022^26^ focused on the prediction of ALS progression and on explainable AI approaches.

In this context, ALS progression is correlated with the risk of early occurrence of NIV, PEG, or death. Note that NIV and PEG are modeled in BTO following the SKOS data model (see “Usage of the Simple Knowledge Organization System (SKOS)” section). In particular, NIV is an instance of type bto:TherapeuticProcedureType defined in NCIT, while PEG is an instance of type bto:SurgicalType and is defined in SNOMED-CT. For DPP, clinicians are interested in understanding how to predict ALS progression for each patient based on the first six months after the first diagnosis. Therefore, for each patient (class bto:Patient), the dataset contained static variables, e.g, onset date (data property bto:eventStart with domain class Onset), diagnosis date (data property bto:eventStart with domain class bto:Diagnosis), (genotypic) sex (data property bto:sex), genetic mutations (class bto:GeneticTesting), and smoking habits (class bto:Smoking). For predicting ALS progression, data about visits for each patient based on the first six months after the first diagnosis is crucial. Thus, BTO models ALSFRS-R and pulmonary function tests and it allows for storing information about the patient who underwent the visit and the date and results of such a visit. Pulmonary function tests (class bto:PulmonaryFunctionTest) report information about the relative FVC (data property pulmonaryFVCRel), while ALSFRS-R questionnaires (class bto:ALSFRS) comprise the ALSFRS-R score (data property bto:revisedALSFRS), all relevant subscores and the score of each item in the questionnaire. Such information is stored as data properties with domain class bto:ALSFRS.

Concerning XAI, ontologies are semantically rich and contextualized resources that end users can easily understand – thus being suited to support XAI approaches [70]. BTO has been used to compare three model-agnostic, post-hoc explainability methods (SHAP, LIME, and AraucanaXAI) [71]. All these methods provide, as explanations, the variables (and associated values) that motivate a certain AI outcome. XAI methods were evaluated in terms of identity, fidelity, separability, and time, but found no definitive superior performance. Nunes et Al. [6] propose a new approach to generate semantic similarity-based explanations for patient-level predictions starting from BTO, using five steps: (1) enrich BTO by integrating additional biomedical ontologies; (2) if not available, annotate patients semantically; (3) compute the similarity between patients; (4) select patients whose progression motivates a specific prediction; and (5) visualize the generated explanations. Details about the developed approaches and results for each task are available in each participant’s paper and in the iDPP@CLEF 2022 overview [4, 5].

iDPP@CLE 2023 (second edition)^27^ focused on the prediction of MS progression and on analyzing the impact of exposition to pollutants on predicting ALS progression.

In this context, MS worsening is defined based on the increase in the EDSS score. Thanks to the KB built accordingly to BTO, static data about MS patients and 2.5 years of visits can be easily extracted. About visits, BTO can provide information about relapses (class bto:Relapse), EDSS (class bto:EDSS), EPs (class bto:EvokedPotentials), and MRIs (class bto:MRI). In particular, useful information can be the start date of relapses (data property bto:interventionStart with domain class bto:Relapse), the EDSS date (data property bto:interventionStart with domain class bto:EDSS), the EDSS score evaluated by the clinician (data property bto:clinicallyEvaluatedEDSS), and the type of MS observed together with the date of the observation (data properties bto:multipleSclerosisType and bto:interventionStart with domain class bto:ClinicalEvaluation). About EPs, BTO provides the date (data property bto:interventionStart with domain class bto:EvokedPotentials), whether the EP response was normal or altered (bto:evokedPotentialsResponse), the type of EP performed (bto:evokedPotentialsType), and the body area of the exam (bto:evokedPotentialsLocation). Finally, MRIs information comprises the date of the exam (data property bto:interventionStart with domain class bto:MRI), the area in which the MRI was performed (object property bto:anatomicalLocation with range class bto:AnatomicalSite) and whether the test observes some lesions in T1 or T2 (data properties bto:lesionsT1MRI and bto:lesionsT2MRI).

iDPP@CLEF 2023 integrates environmental data for predicting ALS progression, by providing air pollutants concentration collected by sensing devices in different locations. Such measurements are in raw format, i.e., value registered by the sensors, and calibrated by removing the seasonal component and noise. Pollutants information comprises several pollutants, e.g., SO2, O3, PM10, PM2.5, and CO. As described in “Environmental data” section, pollutants’ concentrations are modeled with a subclass of pollution:Air_pollution_concentration, e.g., pollution:PM10_concentration, and the concentration measures are stored with two data properties, namely bto:concentrationMeasurement and bto:measurementCaligrated.

More recently, BTO has also been used to model prospective data within the iDPP@CLEF 2024 challenge [68, 69]. More in detail, in this challenge, patients’ profiles were extended with wearable sensors’ data that could be used to predict the progression. To do so, we extended the current ontology with a class called bto:Wearable Data Measurement and a series of subclasses that allowed us to model all the sensors’ data we had access to^28^.

Other relevant applications where BTO could be integrated as a core component are manual text annotations, where experts annotate text using the concepts associated with a reference ontology [72], and automatic knowledge extraction, where automated systems extract information from unstructured text and normalize it against reference ontologies [73, 74].

## Ontology deployment

We deployed BTO for all iDPP@CLEF challenges. To prepare the data for these challenges, we ran several SPARQL queries on the BTO-based KB, from which we extracted anonymized patient data containing the required information. We report some SPARQL queries that could be used to prepare such datasets showing useful use cases for BTO. For instance, we report how static variables can be extracted (Query 1), smoking habits (Query 2), EP responses for MS patients (Query 3), ALSFRS-R for ALS patients (Query 4), and how pollutants exposure is encoded inside BTO (Query 3). For each query, we report a table with some query result samples. Note that the displayed data is synthetic to avoid releasing any patients’ sensitive information.

Query 1 displays static variables, i.e., information about the patients that do not change over time. For instance, we return the patient’s biological sex assigned at birth, ethnicity, and date of diagnosis. We also report the age at the onset, i.e., the patient’s age when the first symptoms occurred, and the place area, i.e., the urban classification of the patient’s birthplace. Table 10 reports some examples of the query result. 
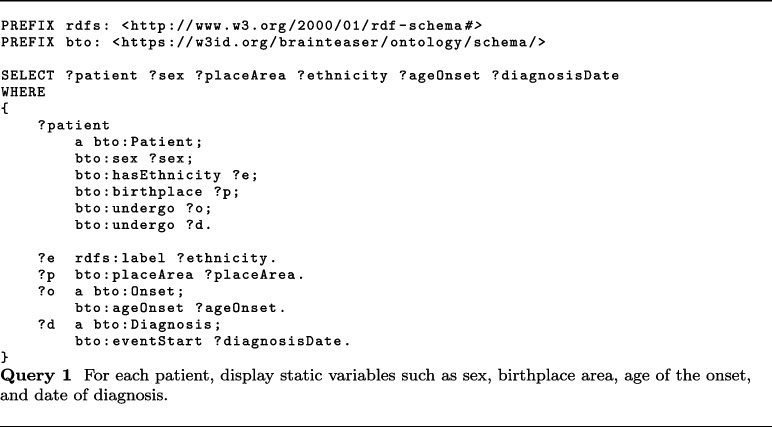

**Table 10:** Result sample for Query 1: “For each patient, display static variables such as (genotypic) sex, birthplace area, onset age, and diagnosis date”. Columns are named as the selected variables in the query

patient	sex	placeArea	ethnicity	ageOnset	diagnosisDate
74wb4y7	Male	Rural	Caucasian	26	05-02-2018
e22tk3i	Female	City	Hispanic	59	12-07-2015
...	...	...	...	...	...
r1o0qtb	Male	Town	Caucasian	67	09-11-2021

Smoking has emerged as a possible environmental risk factor for MS, which can increase the risk of MS development and can accelerate the disease progression [75, 76]. On the other hand, the correlation between smoking and ALS is controversial. Some studies assert that smoking is a risk factor for ALS development, mortality, and disease progression [77, 78], while others found no association between the two [79]. In this regard, we collect data about patients’ smoking habits to investigate a possible correlation between smoking and ALS or MS. For instance, BTO encodes information on how smoking habits could influence ALS progression by increasing the likelihood of NIV or PEG interventions or even hasten death. BTO models the year a patient started or stopped smoking, the number of daily cigarettes, and whether one started smoking before age 20. In addition, we provide a data property called “packYear” which quantifies the lifetime tobacco exposure and is defined as $(daily\_ cigarettes)*(smoking\_ years)/20$, where “daily_cigarettes” is the number of cigarettes smoked in a day and “smoking_years” is the number of years in which a patient smoked. Query 2 returns all patients in the KB built following BTO who smoke or smoked in the past. In particular, we return the patient’s identifier, the year each patient started smoking, and, if present, the year each patient quit. Table 11 reports some examples of the query result.
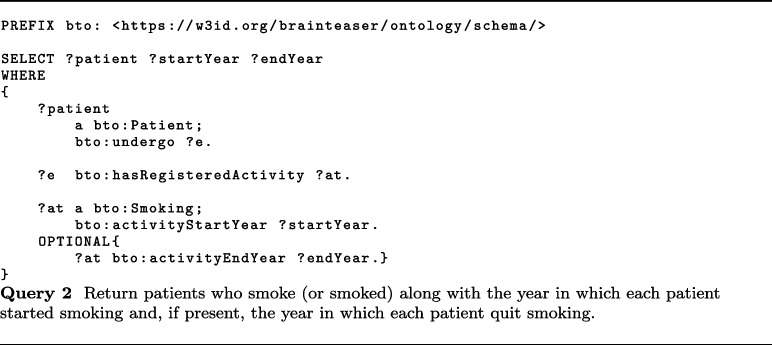

**Table 11:** Result sample for Query 2: “Return patients who smoke (or smoked) along with the year in which each patient started smoking and, if present, the year in which each patient quit smoking”. Each column is named after the selected variables in the query. Symbol “–” denotes missing values

patient	startYear	endYear
patient-74wb4y7	1998	2019
patient-e22tk3i	2003	–
...	...	...
patient-r1o0qtb	2002	2021

Evoked Potentials (EP) measure neuro-electric responses that are useful to monitor changes in MS patients’ neurological status [80]. EPs key information regards whether the test response was normal or altered and the area of the exam, e.g., left, right, upper right, or lower right. Query 3 returns the latest EP for each patient, along with information about the EP type, response, and interested location. Table 12 reports some results samples.
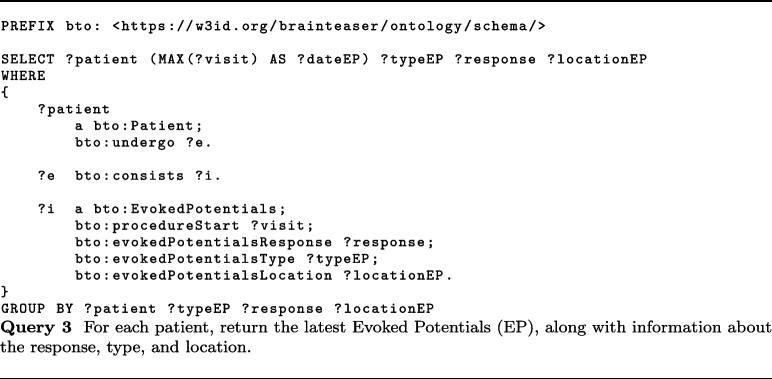

**Table 12:** Result sample for Query 3: “For each patient, return the latest Evoked Potentials (EP), along with information about the response, type, and location”. Each column is named after the selected variables in the query

patient	dateEP	typeEP	response	locationEP
mlgtr7m	20-05-2022	Motor	Altered	Lower Left
374cfp4	13-10-2021	Visual	Normal	Right
...	...	...	...	...
5biezan	28-07-2020	Auditory	Normal	Left

The ALS Functional Rating Scale (ALSFRS) monitors the progression of disability in patients with ALS. The revised ALSFRS (ALSFRS-R) maintains the properties of the original scale and incorporates additional assessments of respiratory functions, i.e., dyspnea, orthopnea, and the need for ventilatory support [51]. ALSFRS-R measures 12 physical functions with scores between 4 and 0, where lower values denote more pronounced impairments. Several subscores, such as the bulbar, motor, and respiratory subscores, can be computed. Query 4 returns all visits where the ALSFRS-R has been assessed and the corresponding patient. Besides the ALSFRS-R, we also return the bulbar, motor, and respiratory subscores. Table 13 reports some result samples.
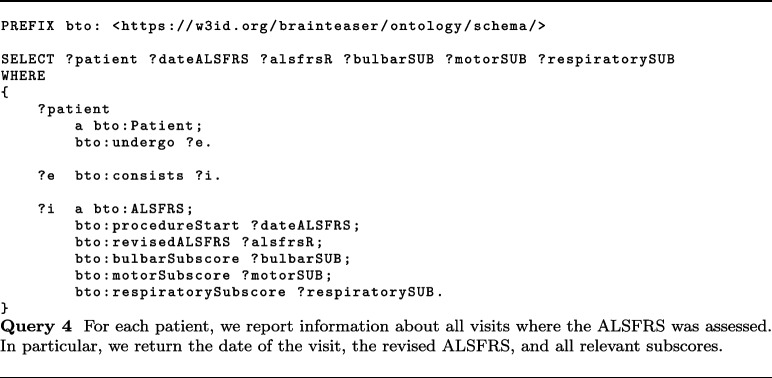

**Table 13:** Result sample for Query 4: “For each patient, we report information about all visits where the ALSFRS was assessed”. Each column is named after the selected variables in the query. “resp. SUB” stands for respiratorySubscore

patient	dateALSFRS	alsfrsR	bulbarSUB	motorSUB	resp. SUB
uxnin8h	29-06-2021	6	2	2	2
hsuwr8i	23-01-2020	27	7	8	12
hsuwr8i	15-06-2019	20	5	3	12
...	...	...	...	...	...
1ea3pvv	15-11-2017	29	11	7	11
ak7kadv	22-02-2012	43	20	12	11

BTO integrates environmental data with patients’ positional information so that a possible relationship between MS or ALS and pollutants can be investigated. To this end, Query 5 reports the PM10 concentration levels to which each patient has been exposed. In particular, for each patient, we report the timestamp of the pollutant detection and the two measured values. “measuredValue” is the concentration level measured by the sensor, while “decomposedMeasurement” is the measured value with seasonal component and noise removed. Pollutant concentrations are linked to each patient by means of the residence information. Thus, we filter the detected concentrations and keep only the measurements obtained when the patient lived in the specified place. For instance, if a patient lived in Rome from 1998 to 2013 we keep pollutant concentrations registered in Rome from 1998 to 2013 and filter out the rest. Table 14 reports some examples of the query result.
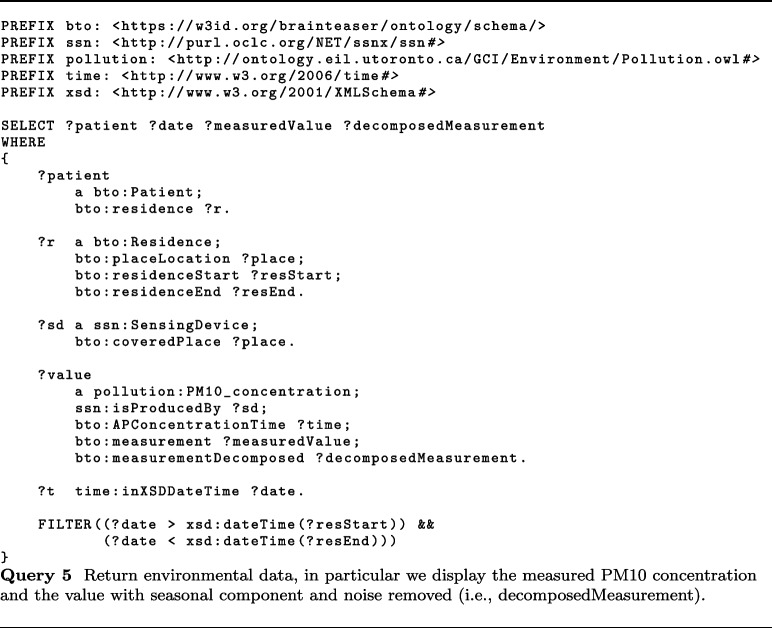

**Table 14:** Result sample for query 5. Each column is named after the selected variables in the query. “dec. Meas.” stands for decomposedMeasurement

patient	detectionDate	measuredValue	dec. Meas.
rogunhr	2017-04-06	6.9	23.43
ymmd3m2	2022-08-14	16.0	24.82
...	...	...	...
ak7kadv	2014-07-17	36.0	22.16

## Conclusion

This work presents the development of BTO, an ontology that provides a unifying data structure and ontologically models clinical data concerning patients affected by ALS and Multiple Sclerosis (MS). To favour its adoption, BTO has been designed following the OBO design principles and FAIR principles. BTO has been validated via several automatic tools, as well as according to the expertise in the medical domain by several medical experts. BTO is based on eight semantic areas, describing different types of occurrences and events that might happen during the progression of the above-mentioned neurological diseases. These semantic areas include information about the demographic of the patients, as well as medical procedures that they might undergo, such as diagnostic procedures or therapeutic ones.

BTO represents a novel semantic resource under a number of different aspects. Firstly, it relies on an event-based representation of the clinical events. This makes it particularly versatile and suitable for modeling several diseases beyond ALS and MS. Secondly, It is one of the first resources dealing in a systematic manner with the ALS disease – bringing forward the state of the art in this regard. Thirdly, while previous endeavours in modeling MS exist, none of them embedded aspects related to the clinical progression of the disease. In this regard, BTO is among the first in allowing to put in relation the MS disease, with the clinical history of affected patients. Finally, it is the only ontology designed, in this specific domain, to also include environmental details.

The development of BTO required a thorough analysis of the source data and iterative feedback from clinicians. This process improved the original data and the data collection process, as well as the identification of additional relevant aspects.

Future works include the extension of BTO to other rare brain-related diseases, such as Parkinson’s and Alzheimer’s diseases, as well as the integration of other multimodal data and the linkage between neurological diseases and gut disorders – which emerging evidence hints it may play a critical role in neurological diseases like MS, Parkinson’s disease, and Alzheimer’s disease [81]. Moreover, the BTO and future extensions will be employed in entity and relation extraction tasks as well as in link prediction tasks, particularly useful in the ever evolving biomedical domain.

The realm of neurological diseases, especially when considering the gut-brain interplay, is vast, complex, and heterogeneous. The BTO is not the end of the journey but rather the starting point for a comprehensive modeling effort of this domain. It can be used as a common foundation to extend to other diseases as well as to further elaborate on ALS and MS.

## Data Availability

The BTO ontology is available at the following link https://zenodo.org/records/7886998 as well as in multiple ontology aggregators such as NCBO (https://bioportal.bioontology.org/ontologies/BT-ONTOLOGY).

## Abbreviations

AI: Artifical Intelligence
ALS: Amyotrophic Lateral Sclerosis
ALSFRS-R: Revised Amyotrophic Lateral Sclerosis Functional Rating Scale
ATC: Anatomical Therapeutic Chemical Classification
BMI: Body Mass Index
BS: Brier Score
BTO: BrainTeaser Ontology
CDE: Common Data Element
CLEF: Conference and Labs of the Evaluation Forum
CNS: Central Nervous System
CO: Carbon Monoxide
CSF: Cerebrospinal Fluid
CUI: Concept Unique Identifier
DPP: Disease Progression Prediction
EDSS: Expanded Disability Status Scale
EP: Evoked Potentials
ESCO: European Skills, Competences, qualifications and Occupations
ETL: Extract, Transform, Load
FAIR: Findable, Accessible, Interoperable, Reusable
FOAF: Friend-Of-A-Friend
FTD: Frontotemporal Dementia
FVC: Forced Vital Capacity
HORD: Holistic Ontology of Rare Diseases
ICD9CM: International Classification of Diseases, Version 9 - Clinical Modification
ICD10: International Classification of Diseases, Version 10
iDPP@CLEF: intelligent Disease Progression Prediction Challenge at CLEF
IE: Information Extraction
IRI: Internationalized Resource Identifier
KB: Knowledge Base
KINGS: King’s clinical staging method
MedDRA: Medical Dictionary for Regulatory Activities Terminology
MHO: Mental Health Ontology
MiToS: Milano-Torino functional staging system
MRI: Magnetic Resonance Imaging
MS: Multiple Sclerosis
MSPD: Multiple Sclerosis Patient Data Ontology
MSO: Multiple Sclerosis Ontology
NCIT: National Cancer Institute Thesaurus
NDO: Neurological Diseases Ontology
NIV: Non-Invasive mechanical Ventilation
NLP: Natural Language Processing
NO2: Nitrogen Dioxide
OAE: Ontology of Adverse Events
OBDA: Ontology-Based Data Access
OBO: Open Biological and Biomedical Ontology Foundry
RDF: Web Ontology Language
O3: Ozone
PEG: Percutaneous Endoscopic Gastrostomy
PM: Particulate Matter
PM10: Particulate Matter <10 *µ*m
PM2.5: Particulate Matter <2.5 *µ*m
RDF: Resource Description Framework
RDFS: RDF Vocabulary Definition Language
RO: Relations Ontology
SKOS: Simple Knowledge Organization System
SNOMED-CT: Systematised NOmenclature of MEDicine Clinical Terms
SO2: Sulphur Dioxide
STMSO: Symptomatic Treatment of Multiple Sclerosis Ontology
UISS: Universal Immune System Simulator
UMLS: Unified Medical Language System
URI: Unique Resource Identifier
XAI: eXplainable AI

## Footnotes

1 https://zenodo.org/records/7886998

2 https://bioportal.bioontology.org/ontologies/BT-ONTOLOGY

3 https://archivo.dbpedia.org/info?o=https://brainteaser.dei.unipd.it/ontology/schema/bto.owl

4 http://purl.obolibrary.org/obo/ncit.owl

5 https://www.nlm.nih.gov/healthit/snomedct/index.html

6 https://ec.europa.eu/esco/lod/static/model.html

7 https://bioportal.bioontology.org/ontologies/ATC

8 https://bioportal.bioontology.org/ontologies/OAE

9 http://ontology.eil.utoronto.ca/GCI/Environment/Pollution.owl

10 https://www.nlm.nih.gov/research/umls/index.html

11 https://bioportal.bioontology.org/ontologies/ICD9CM

12 https://bioportal.bioontology.org/ontologies/ICD10

13 https://bioportal.bioontology.org/ontologies/MEDDRA

14 https://obofoundry.org/principles/fp-000-summary.html

15 https://www.go-fair.org/fair-principles/

16 https://oops.linkeddata.es/index.jsp

17 http://iot.ee.surrey.ac.uk/SSNValidation/

18 https://www.w3.org/RDF/Validator

19 http://graphite.ecs.soton.ac.uk/checker/

20 https://archivo.dbpedia.org/info?o=https://brainteaser.dei.unipd.it/ontology/schema/bto.owl

21 https://archivo.dbpedia.org/rating

22 https://bioportal.bioontology.org/ontologies/BT-ONTOLOGY

23 https://uts.nlm.nih.gov/uts/umls/home

24 https://www.w3.org/TR/2009/REC-skos-reference-20090818/

25 http://ontology.eil.utoronto.ca/

26 http://brainteaser.dei.unipd.it/challenges/idpp2022/.

27 http://brainteaser.dei.unipd.it/challenges/idpp2023/.

28 BRAINTEASER Deliverable 9.6: “Shared data package for the evaluation challenge and integration with EOSC” [Technical Report]

## Data availability

The brainteaser ontology can be accessed at https://zenodo.org/records/7886998.
